# A Case of a Metastatic Pancreatic Neuroendocrine Tumor: A Surgical Conundrum Wrapped in Functionality's Embrace

**DOI:** 10.7759/cureus.56893

**Published:** 2024-03-25

**Authors:** Adam Mylonakis, Michail Vailas, Panagiotis Sakarellos, Lysandros Karydakis, Eleandros Kyros, Spyridon Davakis, Alexandros Papalampros, Evaggelos Felekouras

**Affiliations:** 1 First Department of Surgery, Laikon General Hospital, National and Kapodistrian University of Athens, Athens, GRC

**Keywords:** gastrinoma, secondary insulinoma, liver neoplasm, neuroendocrine tumor, pancreatic neoplasm

## Abstract

This case study reports a rare case of a non-functioning metastatic pancreatic neuroendocrine tumor (pNET) transforming into a functioning pNET. A 59-year-old male, previously treated with distal pancreatectomy, splenectomy, lymph node dissection, liver metastasectomy, and pharmacotherapy, presented with weakness, hypoglycemia, and daily episodes of watery diarrhea. A functioning neuroendocrine liver metastasis expressing insulin and gastrin was identified. Surgical intervention, including left lateral hepatectomy and microwave ablation of multiple intrahepatic lesions, resulted in symptom resolution and uneventful recovery. However, metastatic liver disease re-emerged seven months post-surgery, necessitating chemotherapy. This case highlights the importance of vigilance for symptom development in non-functioning pNETs, signaling potential disease relapse and phenotype transformation, and suggests surgical treatment as a viable option in select cases.

## Introduction

Pancreatic neuroendocrine tumors (pNETs) are a subgroup of neuroendocrine neoplasms (NEN) originating from endocrine cells of the pancreas. They exhibit unique biological behavior and require clinical approaches that differ significantly from those used for pancreatic adenocarcinoma [1].

Based on the clinical evidence of syndromes caused by excessive tumor hormone secretion, pNETs are divided into two types, namely, functioning and non-functioning. Functioning pNETs, comprising about 35% of pNETs, are further categorized based on the specific hormones they secrete, including insulinomas, gastrinomas, and glucagonomas, among others [2]. Non-functioning pNETs, on the other hand, do not secrete hormones or produce clinical symptoms related to hormone hypersecretion. It is extremely rare, but documented, that non-functioning pNETs can transform into functioning pNETs. However, the mechanism underlying this transformation remains unclear and is a subject of ongoing research.

In this study, we report the case of a 59-year-old male patient who was referred to our Surgical Department for the management of a functioning pNET. This presentation occurred nine years after the surgical resection of a previously diagnosed non-functioning, metastatic pNET. Additionally, we provide a review of our considerations regarding this rare clinical scenario.

## Case presentation

A 59-year-old man was referred to our Surgical Department due to daily episodes of watery diarrhea and multiple hypoglycemia episodes leading to syncope, resulting in five hospital admissions over the previous year. His medical history revealed that he had undergone a distal pancreatectomy, splenectomy, lymph node dissection, and liver metastasectomy for a non-functioning pNET nine years prior to the current hospitalization. The tumor was then pathologically staged as pT3N1M1 with a Ki-67 index of 4%. Postoperatively, the patient initially received somatostatin analogs for two years, which were later switched to everolimus due to progressive liver disease. However, everolimus treatment was discontinued due to intolerance, and the patient was subsequently treated with peptide receptor radionuclide therapy (PRRT). He underwent four cycles of PRRT two years after the initial diagnosis, followed by an additional four cycles six years post-diagnosis.

**Figure 1:**
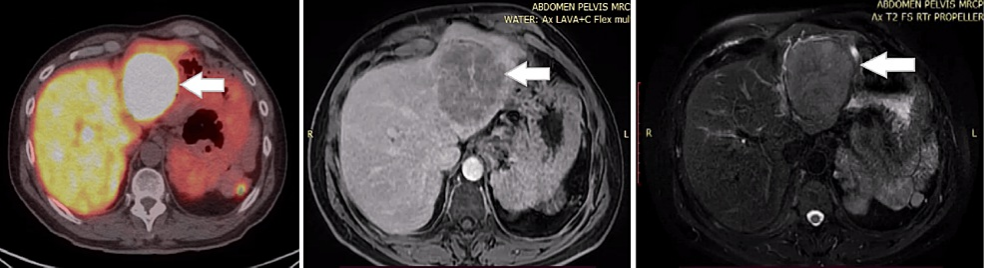
Axial PET-CT and MRI images of the abdomen The white arrow demonstrates a left lobe liver lesion. PET-CT: positron emission tomography-computed tomography; MRI: magnetic resonance imaging

Upon admission, the diagnosis of a relapsed functioning pNET was highly suspected based on laboratory findings. The gastrin level was 41.1150 pg/ml (normal range <110 pg/ml), and chromogranin A (CgA) was markedly elevated at 210 nmol/l (normal range <4 nmol/l). Due to protracted hypoglycemia, the patient was treated with octreotide, diazoxide, and a continuous infusion of dextrose in water (D/W) at 35%. Upper gastrointestinal (GI) endoscopy revealed multiple ulcerations in the gastric and duodenal mucosa. Additionally, a colonoscopy was performed and ruled out other primary sites. Magnetic resonance imaging (MRI) of the abdomen identified a significant 8.1 cm lesion in the left lobe of the liver and multiple smaller lesions in liver segments VI and VII. A 68Ga/64Cu-DOTA-somatostatin analog (SSA) positron emission tomography-computed tomography (PET-CT) scan demonstrated increased uptake in the liver, epiphrenic lymph nodes, and the right thyroid lobe. The diagnostic workup also included an MRI scan of the brain, which revealed the presence of a non-functioning pituitary adenoma (Figure 1).

**Figure 2:**
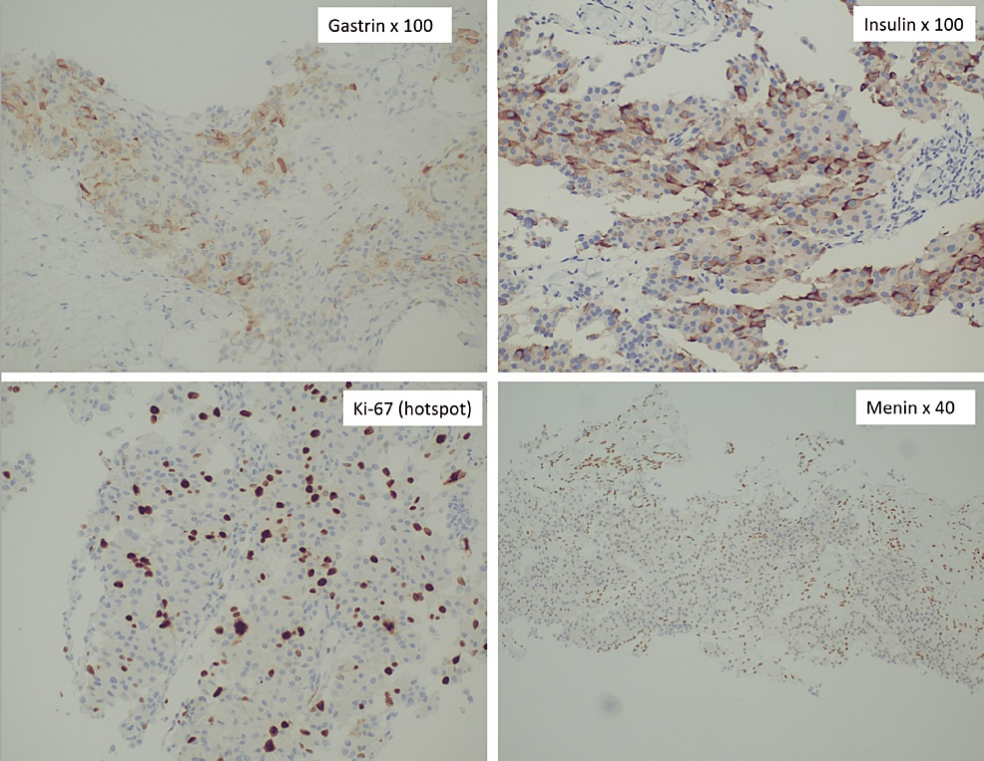
Immunohistochemical staining of liver biopsy for gastrin (x100), insulin (x100), Ki-67 (hotspot x100) and menin expression (x40)

Following a multidisciplinary team review, a percutaneous liver biopsy of the left lobe of the liver was performed under ultrasound guidance. The histological analysis identified a neuroendocrine tumor while immunohistochemical staining demonstrated strong-diffuse insulin expression and moderate-diffuse gastrin expression. The Ki-67 proliferation index was 31%, leading to the classification of the tumor as a Grade 3 neuroendocrine tumor (NET G3) while menin was not expressed in the tumor cells (Figure 2).

Due to the persistence of symptoms indicative of a functioning pNET and resistance to medical therapy, the patient was referred for surgical intervention. No intervention was planned for the epiphrenic lymph nodes or the thyroid lesion identified through imaging, with the surgical efforts concentrated on areas most significantly contributing to the patient's symptomatic hypoglycemia and diarrhea. He underwent a left lateral hepatectomy with intraoperative ultrasound guidance, resection of a lesion in segment V, and microwave ablation of multiple remaining intrahepatic lesions. Intraoperatively, the patient’s glucose levels normalized, eliminating the need for intravenous infusion of dextrose-containing fluids. Postoperatively, the patient experienced cessation of diarrhea and both gastrin and glucose levels normalized. He made an uneventful recovery and was discharged on the eleventh postoperative day with prescriptions for a proton pump inhibitor and somatostatin analog therapy.

Pathological examination of the resected liver specimens revealed metastases from well-differentiated pNET with two distinct histological patterns. The lesion from segment V showed a Ki-67 proliferation index of 3%, classifying it as a Grade 2 neuroendocrine tumor (NET G2). The tumor from the left liver lobe exhibited a Ki-67 index of 25%, classifying it as a Grade 3 neuroendocrine tumor (NET G3).

**Figure 3:**
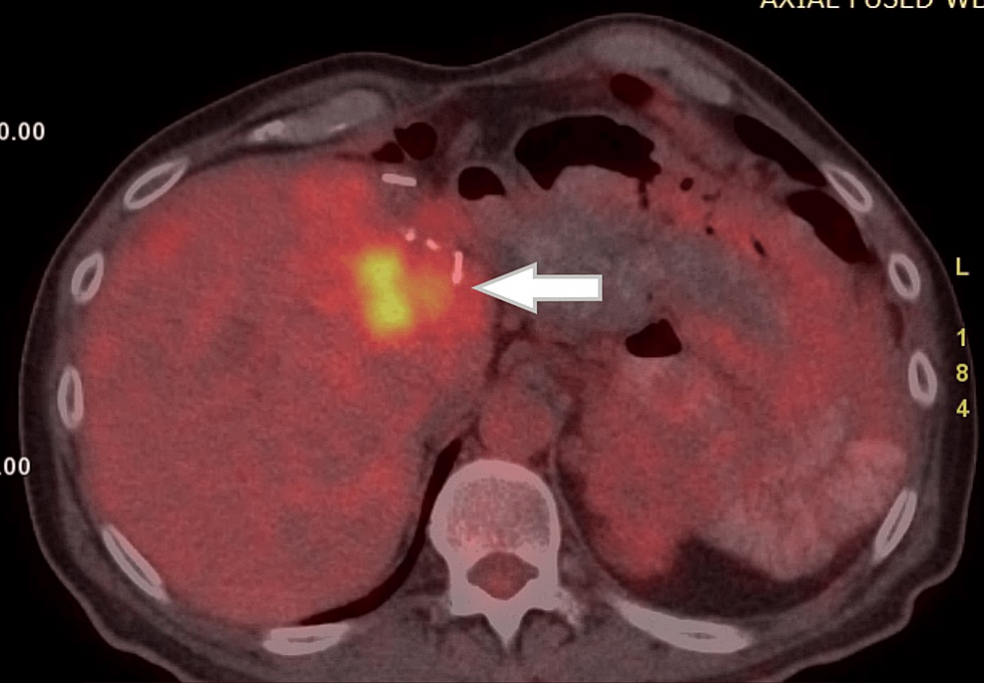
Axial PET-CT image of the abdomen The white arrow demonstrates liver disease recurrence. PET-CT: positron emission tomography-computed tomography

The patient was scheduled for a series of follow-up PET-CT scans. Seven months after the hepatectomy, a new hepatic disease was detected (Figure 3). Subsequently, the patient was treated with a combination therapy of the CAPTEM regimen (capecitabine 750 mg/m^2^ per os twice daily on days 1-14, temozolomide 200 mg/m^2^ per os daily on days 10-14, repeated every 28 days), and somatostatin analogs (SSAs). As of five years post-hepatectomy, the patient remains alive with stable hepatic disease and no evidence of a functioning neuroendocrine syndrome. 

## Discussion

NETs are epithelial neoplasms with predominant neuroendocrine differentiation. Their recognition has increased due to advancements in diagnostic technologies and heightened awareness of this rare entity [3]. Specifically, pancreatic NETs (pNETs) have a clinical detection rate of approximately 1 in 100,000 people, accounting for 1-2% of pancreatic neoplasms [4]. pNETs are classified into two types, functioning and non-functioning, based on the presence or absence of clinical syndromes resulting from excessive hormone secretion by the tumor cells.

While rare, there have been documented cases where non-functioning pNETs transform into functioning tumors [5-8]. This phenotypic change is often associated with liver metastasis. The mechanisms behind this transformation are not fully understood, but hypotheses include the influence of the liver tissue microenvironment, drug-induced changes [5], or the existence of a pluripotent progenitor cell [9].

The primary curative treatment for pNETs, when feasible, is surgical resection. At diagnosis, 40-80% of patients present with metastatic disease, predominantly in the liver (40-93%), followed by bone (12-20%) and lung involvement (8-10%) [10]. Treatment options for metastatic disease are broadly categorized into surgical resection, liver-directed therapies, radionuclide therapy, and medical therapy.

In patients with metastatic disease, surgical intervention is often pursued to relieve symptoms caused by excessive hormone secretion and to improve quality of life [11]. Tumor debulking has also been shown to potentially enhance overall survival in selected cases [12]. Liver-directed therapies include techniques such as ablation, transarterial chemoembolization, or radioembolization [13]. Radionuclide therapy involves the use of radiolabeled somatostatin analogs to deliver targeted radiation to pNETs expressing somatostatin receptors, offering a high therapeutic index [14]. Finally, medical therapy, which may include targeted therapy or chemotherapy in combination with somatostatin analogs, remains a viable option for patients with metastatic NETs [15].

In this report, we discuss a patient with a metastatic non-functional pNET. Initial treatment involved surgery (distal pancreatectomy, splenectomy, lymph node dissection, liver metastasectomy) and medical treatment escalating from a somatostatin analog to everolimus, then to peptide receptor radionuclide therapy (PRRT). Nine years later, the tumor became symptomatic and functional, causing severe hypoglycemia and diarrhea. A left lateral hepatectomy, metastasectomy in liver segment V, and microwave ablation were performed, leading to symptom remission. Seven months post-surgery, a PET-CT scan revealed recurrent liver disease, treated with capecitabine and temozolomide. Despite the recurrence of hepatic disease, the patient displayed no evidence of clinical syndromes related to the neuroendocrine tumor post-resection.

The distinctive aspect of this case lies in the transformation of a metastatic non-functioning pancreatic neuroendocrine tumor (pNET) into a functioning tumor, occurring nine years post the initial diagnosis. Such cases have been rarely documented and are often associated with new or concurrent distant metastases. Notably, this case represents the fifth case, to our current knowledge, where a patient has undergone liver metastasectomy or ablation as a successful therapeutic measure to alleviate symptoms associated with this condition [6-8,16].

Another remarkable trait of this case is the simultaneous expression of two hormones by the tumor, leading to two distinct clinical syndromes: severe hypoglycemia and gastrin-related symptoms. Notably, both syndromes regressed following the surgical resection of the liver metastases. This dual hormone expression and the subsequent effective surgical resolution of the resulting clinical syndromes add further uniqueness to this already rare clinical presentation and underscore the importance of continual monitoring and adaptability in treatment strategies for these patients.

## Conclusions

The delayed transformation of a non-functioning metastatic pNET into a functioning pNET represents an exceedingly rare clinical entity. The emergence of symptoms in patients with previously non-functioning pNETs should prompt a clinical suspicion of disease relapse and potential phenotypic transformation. Based on the findings from this case, we propose that surgical treatment could be a viable consideration in similar scenarios, considering the patient's clinical profile, the severity of symptoms, and the expertise of the treating physician.
